# Contribution of dehydration to END in acute ischemic stroke not mediated via coagulation activation

**DOI:** 10.1002/brb3.1301

**Published:** 2019-04-25

**Authors:** Zhu Shi, Wei C Zheng, Heng Yang, Xiao L. Fu, Wei Y. Cheng, Wei J. Yuan

**Affiliations:** ^1^ Department of Neurology Dongguan Peoples’ Hospital Dongguan PR China

**Keywords:** dehydration, early neurological deterioration, hypercoagulability, ischemic stroke, thromboelastography

## Abstract

**Objective:**

Dehydration is a risk factor for early neurological deterioration (END) after ischemic stroke, yet the underlying mechanism is unclear. Outbalanced coagulation activation may contribute to ischemia progression, concurrently with dehydration‐induced blood viscosity change. We aimed to investigate whether the contribution of dehydration to END was mediated by blood coagulation activation.

**Methods:**

We retrospectively evaluated consecutive patients presenting with mild or moderate stroke (National Institutes of Health Stroke Scale score ≤14) within 24 hr of onset between Jan 2016 and Dec 2017. Dehydration was defined by a serum nitrogen to creatinine ratio (BUN/Cr) of ≥15 and blood coagulation activity was assessed with thromboelastography (TEG). The correlations between BUN/Cr and TEG parameters were assessed and their relationship in the development of END was analyzed.

**Results:**

Of 244 patients, 64 (26.2%) developed END within 3 days after admission. Patients with END had significantly higher BUN/Cr (19.2 ± 5.7 vs. 15.3 ± 2.9, *p* = 0.008), shorter R and K on TEG test (R: 3.9 ± 1.0 vs. 4.6 ± 1.1, *p* = 0.001; K: 1.3 ± 0.5 vs. 1.5 ± 0.4, *p* = 0.005). Comparison between patients with and without dehydration revealed no significant differences in TEG parameters. Multivariate regression suggested that dehydration status (OR 3.91, 95%CI 2.17–8.67, *p* = 0.008) and shorter R tercile on TEG (OR 3.18, 95% CI 1.23–7.90, *p* = 0.016) were independently associated with END; however, the odds ratio of R for END remained unchanged after adjustment for dehydration status.

**Conclusion:**

Our findings suggested that the contribution of dehydration to END after ischemic stroke was mediated by blood coagulation activation.

## 1 INTRODUCTION

Dehydration is a frequently observed condition among patients with acute ischemic stroke and is probably caused by multiple stroke‐related factors such as physical dependency, dysphagia, and disturbed consciousness. This fluid depletion reduces total plasma volume, undermines cerebral perfusion, and changes blood viscosity, potentially exacerbating the degree of hypoxia and scope of the penumbra. A few observational studies reported a two–fourfold increase in the risk for clinical deterioration in dehydrated patients during the early stage of stroke (Bahouth, Gaddis, Hillis, & Gottesman, 2018; Bhatia, Mohanty, Tripathi, Gupta, & Mittal, 2015; Lin, Yang, et al., 2011). Further study suggested that early hydration therapy could facilitate the development of collateral perfusion (Chang et al., 2016). However, no beneficial effects of hemodilution therapy in acute ischemic stroke have been confirmed in major clinical trials (Chang & Jensen, 2014). Hence, the pathophysiological consequences following dehydration cannot solely be compensated by specific fluid resuscitation therapy; other factors may also contribute to clinical deterioration after stroke.

Previous research demonstrated that dehydration was associated with an increased risk of venous thromboembolism in patients with acute ischemic stroke (Kelly et al., 2004; Kim et al., 2017), indicating that an additional mechanism of dehydration promotes coagulation disturbance. An additional study reported that dehydration significantly increased the risk of ischemic stroke in patients with atrial fibrillation (Swerdel et al., 2017). Dehydration‐induced visceral stagnation, as well as other specific factors such as unbalanced blood coagulation activation, which promotes thrombus formation, may play a part. A recent experimental study showed that after dehydrated feeding for 9 days, mice exhibited an increased level of Von Willebrand factor (vWF), which subsequently contributed to a faster blood clotting tendency. Our previous study found that hypercoagulability was more prominent in patients with early neurological deterioration (END) after stroke onset and that this tendency reflected on thromboelastography (TEG) was significantly associated with END (Shi et al., 2018). Considering all the aforementioned observations, the contribution of dehydration to END may be mediated via activating the coagulation cascade. In this study, we sought to investigate the relationship between dehydration status and hypercoagulability in the early phase of ischemic stroke and further elucidate whether the contribution of dehydration to END is mediated by blood clotting activation.

## 2 METHOD

### 2.1 Study population

This retrospective study was performed on consecutive patients admitted for acute ischemic stroke in Dongguan People's Hospital between January 2016 and December 2017. The inclusion criteria were as follows: (a) first‐ever acute ischemic stroke within 24 hr after symptom onset; (b) 18–80 years of age; (c) mild or moderate stroke on admission assessed by a National Institutes of Health Stroke Scale (NIHSS) score ≤14 (Lindsell et al., 2005; Muchada et al., 2014); and (d) length of in‐hospital stay ≥3 days. The exclusion criteria were as follows: (a) hemorrhagic stroke identified by admission computed tomography (CT) scan; (b) administration of intravenous thrombolysis or/and intra‐arterial thrombectomy treatment; (c) history of anticoagulant drug use at least 7 days prior to admission; (d) coexisting severe systemic diseases, including cancer, chronic obstructive pulmonary disease, gastrointestinal bleeding, or chronic renal insufficiency (creatinine > 2 mg/dl or requiring dialysis). The study protocol was approved by the Institutional Review Board of Dongguan People's Hospital, and each patient or their proxies gave informed consent.

Clinical data were collected from the electronic records of the initial assessment when patients arrived at the emergency department, including demographic characteristics, vascular risk factors, and NIHSS score. Laboratory tests, including blood cell counts, lipid profile, BUN, creatinine and C‐reactive protein measurement and other biochemical variables, were performed within 12 hr after admission. The degree of dehydration status was assessed using the blood urea nitrogen/creatinine ratio (BUN/Cr) because of its ready availability in clinical practice and reliability tested in previous studies (Kim et al., 2017; Li, Yin, Zhou, & Chen, 2017; Liu et al., 2014; Schrock, Glasenapp, & Drogell, 2012; Wu et al., 2017), and we used BUN/Cr ≥ 15 as a cutoff for dehydration. The TEG test was performed with a venous blood sample obtained during the initial 12 hr of admission using kaolin as a coagulation activator on a TEG®5000 Hemostasis Analyzer System (Haemoscope, US). Each TEG test value was recorded from a standard tracing, including reaction time (*R*, seconds), kinetic time (*K*, seconds), angle (*α*, degrees), maximum amplitude (MA, mm), clot strength (*G*, dynes/second), estimated percentage lysis (EPL, %), and LY30 (clot lysis since the decay of MA over 30 min).

### 2.2 END definition

Stroke severity was evaluated with NIHSS score on admission and afterward every morning by the same neurologist. Given that a large proportion of patients enrolled in this study initially presented with relatively low NIHSS scores, we incorporated a more sensitive END definition (Siegler et al., 2013) to raise awareness for timely intervention. Specifically, END was defined as an increase in ≥2 on total NIHSS score, an increase in ≥1 on NIHSS subitems of consciousness (1a–1c) or limb strength (5a–6b), or newly emerged symptoms regardless of total NIHSS score changes. For patients who experienced END, follow‐up MRI was carried out immediately to clarify the causes.

### 2.3 Statistical analysis

All statistical analyses were performed using SPSS 21.0 (IBM SPSS Statistics, Chicago, IL). Continuous variables were presented as the mean ± standard deviation if normally distributed or median (interquartile range, IQR) if skewed; categorical variables were presented as a percentage. Comparison of variables between END and non‐END patients was performed using Student's *t* test, the Mann–Whitney *U*‐test, the chi‐square test, or Fisher's exact test as appropriate. To assess the relationship between dehydration status and blood hypercoagulability, we explored the data at the group level and the individual level. Patients were divided into dehydration and control groups, and baseline characteristics and TEG results were compared. Linear regression analysis was conducted with adjustment for age, sex, DM, and baseline NIHSS scores. To evaluate the effects of variables contributing to the development of END, a logistic regression model was constructed with a special focus on dehydration status and coagulation activity. Potential confounding factors and variables with a *p*‐value < 0.10 in the univariate analysis were further tested in a multivariate‐adjusted model. For all statistical methods, *p*‐values below 0.05 were considered indicative of significance.

## 3 RESULTS

### 3.1 Study population

A total of 244 eligible patients were included in the final analysis. The mean age was 65 ± 13 years, with 182 (74.6%) male participants. Dehydration was identified in 134 (55.2%) patients with BUN/Cr ≧15 at admission. The median interval from symptom onset to admission was 8.2 hr, and 64 (26.2%) patients developed END within 3 days afterward. Follow‐up MRI was carried out in 56 END patients, among whom 35 showed diffusion‐weighted image (DWI) hyperintensity enlargement or new lesions outside the initial region, 19 showed no changes, and three showed hemorrhagic transformation on susceptibility‐weighted imaging. Demographic and clinical data are shown in Table 1. By comparison, patients in the END group had a higher prevalence of diabetes mellitus (DM; 41% vs. 19%, *p* = 0.001), higher median baseline NIHSS scores (5 vs. 2, *p* = 0.001), and higher hs‐CRP levels (5.6 vs. 1.7, *p* = 0.016). Dehydration was more frequently observed in patients experiencing END (67% vs. 51%, *p* = 0.028); specifically, patients with END had a significantly higher mean BUN/Cr ratio (16.7 ± 4.1 vs. 15.2 ± 3.5 mg/dl, *p* = 0.017) and urine specific gravity (1.022 ± 0.001 vs. 1.019 ± 0.001 mg/dl, *p* = 0.002) (Table 1). Additionally, patients with END tended to have shorter R (3.9 ± 1.0 vs. 4.6 ± 1.1 min, *p* = 0.001) and K (1.3 ± 0.5 vs. 1.5 ± 0.4 min, *p* = 0.005), indicating faster clotting activation. No significant differences were detected in any TEG parameters, including indicators from clotting formation (*R* time, *K* time, angle), platelet function (MA, *G*), or lysis processes (EPL%, LY30%) at the group level (Table 2).

**Table 1:** Comparison between END and non‐END groups with respect to demographics, baseline characteristics, laboratory, and TEG tests

	END (*n* = 64)	Non‐END (*n* = 180)	*p*
Age, years ± *SD*	66 ± 13	64 ± 13	0.305
Sex, Male, %	46 (72)	146 (81)	0.137
Time from onset hours, median (IQR)	6.8 (3.0–17.6)	9.2 (5.1–19.8)	0.134
Admission NIHSS (points) median (IQR)	5 (3–9)	2 (1–4)	0.001
Risk factors			
Hypertension, *n* (%)	43 (67)	111 (62)	0.432
Diabetes mellitus, *n* (%)	26 (41)	35 (19)	0.001
Dyslipidemia, *n* (%)	19 (30)	53 (29)	0.423
Current smoking, *n* (%)	19 (30)	66 (37)	0.843
Lab tests			
RBC (10^12^/L)	239.0 ± 29.2	235.0 ± 16.3	0.158
Hematocrit	42.3 ± 7.2	40.8 ± 9.4	0.325
Platelet (10^9^/L)	222.2 ± 47.3	231.3 ± 56.0	0.801
Fibrinogen (g/L)	3.9 ± 1.1	3.6 ± 0.8	0.226
Fasting glucose (mmol/L)	7.2 ± 1.3	6.3 ± 0.6	0.490
LDL (mmol/L)	3.2 ± 0.6	3.3 ± 0.3	0.801
BUN (mg/dL)	15.5 ± 4.0	14.6 ± 3.4	0.379
Creatinine (mg/dL)	0.95 ± 0.19	0.96 ± 0.16	0.126
BUN/Cr	16.7 ± 4.1	15.2 ± 3.5	0.017
Dehydration, *n* (%)	43 (67)	91 (51)	0.028
USG	1.022 ± 0.001	1.019 ± 0.001	0.001
hsCRP (mg/L)	5.6 (2.5–7.0)	1.7 (0.7–4.7)	0.016
TOAST			0.300
LAA	35 (55)	91 (51)	
SAD		50 (28)	
CE	8 (13)	14 (8)	
Other/unknown	4 (6)	25 (14)	
Medication			
DAT	45 (70)	120 (67)	0.592
Statin	62 (96.9)	179 (99.4)	0.110

Abbreviations: USG, urine specific gravity; DAT, dual antiplatelet therapy with aspirin and clopidogrel.

**Table 2:** Comparison of TEG values by END and dehydration status

	END *n* = 64	non‐END *n* = 180	*p*	Dehydration *n* = 134	Hydration *n* = 110	*p*
R (min)	3.9 ± 1.0	4.6 ± 1.1	0.001	4.4 ± 1.1	4.6 ± 1.2	0.176
R < 5 min, *n* (%)	51 (79.7)	119 (66.1)	0.042	90 (67.2)	80 (72.7)	0.347
K (min)	1.3 ± 0.5	1.5 ± 0.4	0.005	1.5 ± 0.5	1.5 ± 0.4	0.539
K < 1 min, *n* (%)	9 (14.1)	12 (6.7)	0.071	9 (6.7)	12 (10.9)	0.245
Angle (deg)	68.2 ± 5.7	67.8 ± 5.6	0.099	67.7 ± 5.9	67.6 ± 5.5	0.912
MA (mm)	63.5 ± 6.1	62.6 ± 5.3	0.271	62.4 ± 5.8	62.4 ± 5.3	0.944
G (d/sc)	9,023 ± 2,393	8,648 ± 2092	0.238	8,592 ± 2,176	8,524 ± 2022	0.826
EPL (%)	1.6 (0.6–4.7)	0.7 (0.1–2.4)	0.017	0.9 (0.1–3.0)	1.2 (0.1–3.7)	0.940
LY30 (%)	1.2 (0.2–2.6)	0.2 (0.1–1.7)	0.007	0.5 (0.1–2.4)	0.6 (0.1–2.1)	0.944
A (mm)	58.3 ± 8.6	59.4 ± 7.3	0.304	58.3 ± 7.6	58.5 ± 8.0	0.875
CI	1.9 (0.9–3.1)	1.4 (0.4–2.5)	0.134	1.4 (0.2–2.5)	1.3 (0.5–2.6)	0.917

In the comparison of TEG values between the dehydration group and the nondehydration group, no significant differences were detected at the group level. To further explore the correlation between dehydration status and blood coagulation activity, we performed a multivariate linear regression analysis. After adjusting for age, sex, baseline NIHSS score, and DM history, a linear regression model indicated no significant correlations between the BUN/Cr ratio and each result of the TEG test (*R* time: beta −0.027, *p* = 0.721; *K* time: beta‐0.012, *p* = 0.877; angle: beta 0.091, *p* = 0.216; MA: beta 0.122, *p* = 0.100; *G*: beta −0.096, *p* = 0.192; EPL%: beta 0.055, *p* = 0.471; LY30%: beta 0.084, *p* = 0.263; A: beta −0.139, *p* = 0.066; CI: beta −0.065, *p* = 0.372).

In logistic regression analysis, R ≤ 3.8 min was significantly associated with the development of END (odds ratio [OR] 2.914, 95% confidence interval [CI] 1.428–5.946, *p* = 0.003). Other significant risk factors included dehydration status, female sex, higher baseline NIHSS score, history of DM, and higher serum hs‐CRP level. (Table 3). In the final multivariable logistic regression model, both dehydration status and reduced R terciles were significantly associated with END. However, the odds ratio of reduced R terciles remained unchanged after controlling for dehydration status, suggesting that dehydration status and hypercoagulability separately contributed to the development of END.

**Table 3:** Logistic regression for END

	Model 1		Model 2		Model 3	
	OR 95% CI	*p* value	OR 95% CI	*p* value	OR 95% CI	*p* value
R time						
Low tercile	2.545 (1.147–5.645)	0.022	3.318 (1.233–8.929)	0.018	3.253 (1.202–8.799)	0.020
Median tercile	1.231 (0.538–2.828)	0.623	1.333 (0.508–3.494)	0.559	1.353 (0.541–3.560)	0.540
Reference	—					
Dehydration	2.088 (1.073–4.062)	0.001	2.075 (0.981–2.075)	—		
DM	2.214 (1.102–4.451)	0.001	—			
Baseline NIHSS	1.357 (1.195–1.514)	0.001	—			
Female	3.111 (1.590–6.085)	0.003	—			
hsCRP	1.210 (1.005–1.456)	0.044	—			
Age (10 years)	1.137 (0.911–1.426)	0.237				

Note

Model 1: univariate analysis; Model 2: adjusted for DM, baseline NIHSS score, sex, age, and hsCRP; Model 3: adjusted for dehydration.

## 4 DISCUSSION

Our results suggested that blood hypercoagulability, determined by the reduced R time on TEG, as well as dehydration status with BUN/Cr ≥15, was more prominent in stroke patients with END. Between dehydrated patients and those who were not, however, no significant differences in TEG parameters were detected. Further linear regression analysis showed no significant correlations between the BUN/Cr ratio and TEG parameter. After adjusting for age, sex, DM, NIHSS score and serum hs‐CRP, reduced R, and dehydration status were independently associated with the development of END; furthermore, the odds ratio of R time for END barely changed after adjustment for dehydration status.

END occurs in approximately 10%–30% of acute ischemic stroke patients and is strongly associated with adverse outcomes (Thanvi, Treadwell, & Robinson, 2008). As a clinical operational definition, however, END can actually be caused by distinctive pathophysiological mechanisms. For patients with mild stroke, the extension of symptomatic ischemia into previously asymptomatic oligemic tissue plays the most influential role (Alawneh et al., 2018); for patients with severe stroke, it may also involve vasogenic edema or hemorrhagic transformation. Recent studies revealed that dehydration status was associated with the development of END (Bhatia et al., 2015; Lin, Fann, et al., 2011) and that hydration therapy based on dehydration assessment could prevent END and improve functional outcomes (Lin et al., 2017, 2016). However, the confounding effect regarding distinct END mechanisms in mild or severe stroke was seldom considered in those studies. For severe stroke with a large infarct size, rehydration may adversely exacerbate cerebral edema, causing surrounding tissues to become symptomatic. To this end, our study excluded patients with severe stroke, and the primary END mechanism in this setting may be ischemia progression or reocclusion (Alawneh et al., 2018). Therefore, concluding that the contribution of dehydration to END could be mediated via coagulation activation seems reasonable.

First, we compared TEG parameters during the early stage of stroke between patients with and without dehydration and found no difference. Additionally, we found no relationship between hydration parameters (BUN/Cr ratio) and each TEG parameter. Despite extensive evidence supporting the clinical relevance, the mechanism of how dehydration contributes to ischemia progression immediately after stroke onset remains unclear. Some studies have indicated that hemoconcentration following dehydration contributes to an increase in pivotal factors involved in blood clotting cascades, such as platelets and fibrinogen (Dmitrieva & Burg, 2014). A recent experimental study showed that dehydration feeding for 9 days in mice could trigger an increase in the vWF level and subsequently contribute to blood clotting activation. However, our study did not support the correlation between dehydration and hypercoagulability during the early phase of acute ischemic stroke, indicating that dehydration may play different roles at early or late stages of stroke.

Second, our results suggested that blood hypercoagulability was also significantly associated with END, independent of the detrimental effect of dehydration. Thrombotic tendency has been proposed as another important risk factor for ischemia progression, predisposing the slowed blood flow in microvascular circulation distal to the occluded artery to thrombosis. In fact, some studies reported that dehydration status was negatively associated with the development of collaterality, especially secondary collateral flow through leptomeningeal anastomoses. Although our study did not support the hypothesis that dehydration per se leads to blood hypercoagulability in the early phase of stroke, blood hypercoagulability could also increase the risk of collateral failure by forming small thrombi in oligemic brain tissue. Thus, it may play an independent role in the development of END immediately after stroke onset.

Our study had some limitations. First, this was a retrospective single‐center study, and we enrolled only patients with mild or moderate stroke; selection bias should be taken into consideration while generalizing the results. Second, no gold standard exists for dehydration evaluation. Although BUN/Cr is commonly used to assess dehydration status, it may be influenced by other factors such as acute kidney injury, gastrointestinal bleeding and others that we did not consider in our study. Third, TEG was a coagulation test in vitro, and we used a blood sample from peripheral venous blood; some discrepancy may exist with the cerebral circulation. Finally, our study focused on the early phase immediately after stroke onset, and the correlation between dehydration status and blood coagulability in later stages still needs to be elucidated in further studies.

## 5 CONCLUSIONS

Our findings suggest that dehydration status and blood hypercoagulability are more prominent in patients with END after ischemic stroke and are significantly associated with the development of END. However, our results do not support the hypothesis that the contribution of dehydration to END could be mediated by activating the coagulation cascade. Further studies are warranted to better understand END pathophysiology and will facilitate early awareness of END and improve stroke care.

## CONFLICT OF INTEREST STATEMENT

The authors declare no conflict of interest with this publication.

## Data Availability

The data that support the findings will be available in [clinicaltrial.org] at [https://clinicaltrials.gov/ct2/result?xml:id=]NCT03310931s] following an embargo from the review completion.
